# Fat Grafting in Burn Scar Alleviates Neuropathic Pain via Anti-Inflammation Effect in Scar and Spinal Cord

**DOI:** 10.1371/journal.pone.0137563

**Published:** 2015-09-14

**Authors:** Shu-Hung Huang, Sheng-Hua Wu, Su-Shin Lee, Kao-Ping Chang, Chee-Yin Chai, Jwu-Lai Yeh, Sin-Daw Lin, Aij-Lie Kwan, Hui-Min David Wang, Chung-Sheng Lai

**Affiliations:** 1 Graduate Institute of Medicine, College of Medicine, Kaohsiung Medical University, Kaohsiung, 807, Taiwan; 2 Center for Stem Cell Research, Kaohsiung Medical University, Kaohsiung, 807, Taiwan; 3 Division of Plastic Surgery, Department of Surgery, Kaohsiung Medical University Hospital, Kaohsiung Medical University, Kaohsiung, 807, Taiwan; 4 Department of Surgery, Faculty of Medicine, College of Medicine, Kaohsiung Medical University, Kaohsiung, 807, Taiwan; 5 Department of Anesthesia, Kaohsiung Medical University Hospital, Kaohsiung Medical University, Kaohsiung, 807, Taiwan; 6 Department of Pathology, Kaohsiung Medical University Hospital, Kaohsiung, Taiwan; 7 Department and Graduate Institute of Pharmacology, College of Medicine, Kaohsiung Medical University, Kaohsiung, 807, Taiwan; 8 Department of Fragrance and Cosmetic Science, Kaohsiung Medical University, Kaohsiung, 807, Taiwan; Taipei Medical University, TAIWAN

## Abstract

Burn-induced neuropathic pain is complex, and fat grafting has reportedly improved neuropathic pain. However, the mechanism of fat grafting in improving neuropathic pain is unclear. Previous investigations have found that neuroinflammation causes neuropathic pain, and anti-inflammatory targeting may provide potential therapeutic opportunities in neuropathic pain. We hypothesized that fat grafting in burn scars improves the neuropathic pain through anti-inflammation. Burn-induced scar pain was confirmed using a mechanical response test 4 weeks after burn injuries, and autologous fat grafting in the scar area was performed simultaneously. After 4 weeks, the animals were sacrificed, and specimens were collected for the inflammation test, including COX-2, iNOS, and nNOS in the injured skin and spinal cord dorsal horns through immunohistochemistry and Western assays. Furthermore, pro-inflammatory cytokines (IL-1 β and TNF-α) in the spinal cord were collected. Double immunofluorescent staining images for measuring p-IκB, p-NFκB, p-JNK, and TUNEL as well as Western blots of AKT, Bax/Bcl-2 for the inflammatory process, and apoptosis were analyzed. Fat grafting significantly reduced COX2, nNOS, and iNOS in the skin and spinal cord dorsal horns, as well as IL-1β and TNF-α, compared with the burn group. Moreover, regarding the anti-inflammatory effect, the apoptosis cells in the spinal cord significantly decreased after the fat grafting in the burn injury group. Fat grafting was effective in treating burn-induced neuropathic pain through the alleviation of neuroinflammation and ameliorated spinal neuronal apoptosis.

## Introduction

Burn-related neuropathic pain has been reported in 52% to 67.3% of burn injury patients [1, 2]. Neuropathic pain is a localized sensation of discomfort, such as allodynia and hyperalgesia, and it is difficult to treat with even the most potent analgesic compound [3]. Autologous fat grafting has reportedly improved traumatic neuropathic pain in patients with burn induced neuropathic pain, postmastectomy pain syndrome, and traumatic scar pain [4–6]. The mechanism of fat graft pain relief is unclear. Many hypotheses have been proposed regarding the mechanism of fat grafting such as scar softness, improved scar tissue differentiation, nerve entrapment relief [7], and improved nerve liberation [8]. Klinger et al and Valenti et al also reported that fat grafting reduces scar adherents and similar cushions on the nerve stump [8–10]. Rigotti et al indicated that the fat grafting effect of neuropathic pain may be related to adipose-derived stem cells in fat grafts [11]. Fat grafts and their mesenchymal stem cells have been reported to alleviate inflammation in colitis, stroke, and inflammatory models [12, 13], further prevent second necrosis and apoptosis [14]. A rat model of fat graft alleviated burn-induced neuropathic pain has been established [4]. We used this model to explore the mechanism of fat grafts in improving neuropathic pain.

Pain is processed in a neural network, and the interaction between neurons, microglia, and astrocytes is critical for the initiation and maintenance of chronic pain. Activation of glia cells (eg, microglia and astrocytes) contributes to the pathogenesis of chronic pain through neuron-glial interaction [15, 16]. Evidence increasingly suggests that astrocytes are crucial for promoting and maintaining chronic neuropathic pain and pain sensitization [17]. The activation of astrocytes results in the activation of the nuclear factor κB (NFκB), extracellular regulated kinase (ERK), and Jun N-terminal kinase (JNK) signal pathways[18] and the production of inflammatory mediators, including tumor necrosis factor-α (TNF-α), interlukin-1β (IL-1β), nitric oxide (NO), prostaglandin, and neurotrophins [17]. Inflammation also induces cyclooxygenase-2 (COX-2), causing the sensitization of peripheral nociceptors and generation of pain hypersensitivity, which leads to central sensitization and accompanying chronic pain [15,18,19]. This persistent pain caused by neuroinflammation also triggers neuronal apoptosis [20, 21].

NO, which is synthesized by NO synthase (NOS), is involved in processes related to the regeneration of neuropathic pain [22]. Inhibitors of NOS may have analgesic effects and can be used to treat inflammatory and neuropathic pain [23]. NOS, a key enzyme for neuronal NOS (nNOS) or inducible NOS (iNOS), mediates numerous neuropathic pain symptoms [24]. In the neuropathic pain model, inhibition of NOS diminishes the upregulation of iNOS and nNOS in the in spinal cord and skin. This indicates the efficiency of neuropathic pain alleviation. In this study, we focused on whether fat grafts can regulate cytokines and the downstream target of NO in inflammatory pain and isoforms of NOS, which involved in pain modulation [25].

We hypothesized that autologous fat grafts alleviate burn-induced neuropathic pain through the alleviation of skin inflammation and that neuroinflammation in the spinal cord further diminishes neuron cell apoptosis. The aim of this study was to determine the effect of fat grafts on allodynia and hyperalgesia in burn-induced neuropathic pain. Moreover, we examined hind paw skin inflammatory cytokine expression and NOS. Furthermore; we observed the astrocytic response, iNOS, nNOS, COX-2, B cell lymphoma/lewkmia-2 (Bcl-2) family protein, and apoptosis protein expressions in the spinal cord dorsal horn after fat grafting.

## Materials and Methods

### Animals and experimental design

**Fig 1:**
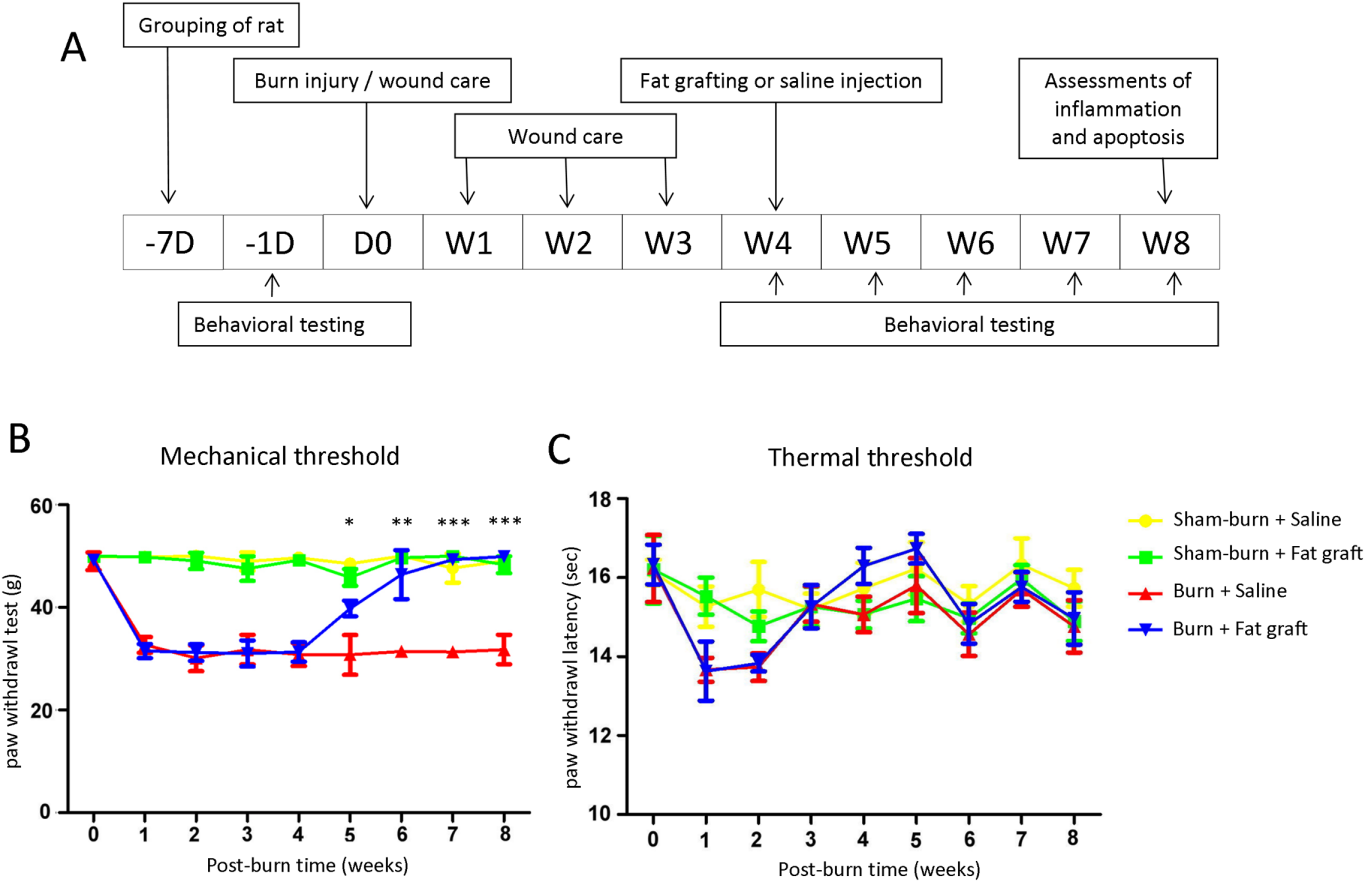
The flowchart of this study and the behavior tests from various groups. (**A**) A flowchart of the study D (day) and W (week). (**B**) The response thresholds to the mechanical stimuli decreased strongly on the burn injury side hind paws in Group C and Group D, 1–4 weeks post burn injury. After the fat grafting, Group D thresholds significantly increased in the following weeks versus Group C. (**C**) The response thresholds to the radiant heat stimuli did not show significant differences between the groups. The sample size was n = 6 for each group. (Data are plotted as mean ± SEM; ***P < 0.001; **P < 0.01; *P < 0.05).

All experiments were performed on adult male Sprague-Dawley rats weighing 180–200 g that were randomly obtained from BioLASCO Taiwan Co., Ltd. All animals were housed in an animal facility at 22°C with a relative humidity of 55% in a 12-hour light–dark cycle with food and sterile tap water available ad libitum. All procedures were approved by the Institutional Animal Care and Use Committee at Kaohsiung Medical University (approval no. 10048). The rats were divided into four groups (n = 6 in each group) as follows: Group A, received saline injections 4 weeks after sham burns (Sham-burn + Saline); Group B, received fat grafts 4 weeks after sham burns (Sham-burn + Fat graft); Group C, received saline injections 4 weeks after burn injuries (Burn + Saline); and Group D, received fat grafts 4 weeks after burn injuries (Burn + Fat graft). The experimental flow chat was presented in Fig 1A.

### Full-thickness burn injury and behavior test

As described in our previous study [4], the third-degree burn was caused by placing the right hind paw on the 75 ± 0.5°C heated metal block in a water bath with a 100 g weight on the hind paw for 10 seconds under anesthesia with Zoletil^®^ 50 (50 mg/kg body weight) (Virbac Laboratories, Carros, France). Silver sulfadiazine cream was applied to the wound for approximately 3 weeks until it healed. Paw withdrawal latency tests (PWLs) and paw withdrawal threshold tests (PWTs) were measured using the same method as our previous study 1 day before the burn injury, 4 weeks after the burn injury and at 1 week intervals for an additional 4 weeks.

These behavioral reactions were measured in the burned hind paw and on the contralateral side. The PWL was used to estimate thermal hyperalgesia. The PWL was recorded using a Plantar Test (Hargreaves Apparatus, Ugo Basile, Varese, Italy). In brief, an infrared radiant heat source was positioned beneath the plantar surface of the hind paw. The time from application of the heat source to withdrawal of the hind paw was defined as the PWL (measured in seconds).

The PWT was recorded using a Dynamic Plantar Aesthesiometer (Ugo Basile, Varese, Italy). The rats were placed on a metal mesh, and a mechanical stimulus was applied using the automated tester with a metal rod (2 mm in diameter) to stimulate the plantar surface of the hind paw. The pressure applied to the rod was increased at a rate of 2.5 g/second until the animal withdrew the paw, which was recorded as the lowest force (g). Each measurement was repeated six times at intervals of 10 minutes with 30 minutes of rest between hind paw applications.

### Autologous fat graft

Four weeks after burn injuries, the left inguinal fat was harvested under Zoletil 50 anesthesia. The adipose tissue was cut into pieces with scissors and aspirated into a 1-mL syringe until 0.4 mL of volume was reached. The fat graft was injected into the subcutaneous area of the burn-injured hind paw skin with a 19-gauge needle. The donor site was closed with nylon 4–0. The control group was injected with 0.4 mL of normal saline in the burn-injured hind paw skin subcutaneously.

### Western blot analysis

Rats were sacrificed 4 weeks after burn injuries by an overdose of Zoletil 50. Lumbar 3,4,5 (L345) spinal cords were collected, and the dorsal horn area and skin were separated, frozen in liquid nitrogen, and stored at -80°C. L345 dorsal horn specimens and skin were homogenized in an ice-cold lysis buffer, T-PER Tissue Protein Extraction Reagent (Thermo Scientific) with one tablet of Complete Protease Inhibitor Cocktail (Roche) per 25 mL and then centrifuged (15,000 g) for 30 minutes at 4°C. Each protein concentration in the supernatant was measured using bovine serum albumin as the standard. The procedures and analyses of Western blots were performed through the same method as our previous report [26]. The primary antibodies of COX-2 (1:1000, Cell Signaling, Danvers, MA), iNOS (1:1000, Abcam, Cambridge, MA), and nNOS (1:1000, Abcam, Cambridge, MA), protein kinase B (AKT) (1:1000, Cell Signaling, Boston, MA), p-AKT (1:1000, Cell Signaling, Boston, MA), Bcl-2 Associated X protein (Bax) (1:1000, Proteintech Group, Chicago, IL), Bcl-2 (1:1000, Abcam, Cambridge, MA), and β**-**actin (1:20000 dilution, Sigma-Aldrich, Saint Louis, MO) were used in this study.

### Immunohistochemistry (IHC) detections of COX-2, iNOS, and nNOS

Hind paw skin was formalin-fixed and embedded in paraffin, and skin of 10 μm thickness was cut and mounted on glass slides, deparaffinized, and rehydrated in graded alcohol solutions. The skin sections were subjected to antigen retrieval by heating the sections to 121°C in 0.1 mol/L of citrate buffer (pH 6.0) in an autoclave for 10 minutes and slowly cooling the sections to room temperature. Furthermore, the sections were incubated for 5 minutes with 3% of H_2_O_2_ to quench the endogenous peroxidase activity. After the blocking of nonspecific sites with 5% goat serum in a phosphate buffered saline (PBS) for 30 minutes, the sections were incubated overnight at 4°C with a rabbit polyclonal antibody against COX-2 (1:200, Cell Signaling, Danvers, MA), iNOS (1:200, Abcam, Cambridge, MA) and nNOS (1:200, Abcam, Cambridge, MA). The sections were then incubated with a secondary antibody conjugated with horseradish peroxidase for 30 minutes at room temperature. Finally, the slides were incubated in 3,3-diaminobenzidine for 5 minutes before undergoing Mayer’s hematoxylin counterstaining for 60 seconds and being mounted.

### Measurement of IL-1β and TNF-α in the spinal cord dorsal horn

The lumbar spinal cord segments were dissected. Spinal cord dorsal horn tissues were homogenized in a lysis buffer. The Bicinchoninic Acid Protein Assay (Pierce) is used to check protein concentrations. And ELISA was performed as in previous studies [26]. The standard curve was included in each experiment. IL-1β and TNF-α levels were measured using enzyme-linked immunosorbent assay kits for the quantitative detection of IL-1β and TNF-α (eBioscience, USA). The reference range for the enzyme-linked immunosorbent assay kit was from 0 to 1400 (IL-1β), and from 0 to 750 (TNF-α) pg/mL. All the measurements were recorded in triplicate.

### Immunofluorescence detection of p-IκB and p-NFκB

The lumbar spinal cord segments were harvested 4 weeks after the fat grafts or vehicle treatments. The fresh-frozen sections were performed through the same method as the previously mentioned immunohistochemistry procedure [4]. For double immunofluorescence staining, the spinal cord dorsal horn was incubated with a mix of polyclonal p-I*κ*B (1:100 dilution, Cell Signaling) and monoclonal NeuN (a neuron cell marker, 1:1000, Millipore, Temecula, CA); polyclonal p-NF*κ*B (1:100 dilution, Cell Signaling, Boston, MA); monoclonal GFAP (an astrocyte marker, 1:1000 dilution, BD Biosciences San Diego, CA); and p-JNK (1:100 dilution, Cell Signaling, Boston, MA) overnight at 4°C. The appropriate secondary antibody conjugated with goat anti-rabbit Cy3 (red, Millipore, Temecula, CA) and goat anti-mouse Alexa Flour 488 (green, Invitrogen, Carlsbad, CA) was added. Images were acquired using a fluorescence microscope (Leica DMI6000).

### Measurement of apoptosis in the spinal cord

The L3 to L5 segments were removed and embedded in an optimal cutting temperature compound to prepare frozen sections cut into 16 mm thick slices. The procedures were mentioned in previous report [26]. Briefly, apoptotic cell death was detected using the TUNEL assay according to the manufacturer’s suggestions (Millipore, ApopTag fluorescein in situ apoptosis detection kit S7110), following incubated with an NeuN primary antibody (1:1000, Merck Millipore, Bedford, MA) at 4°C. The sections were then incubated with a Cy3-conjugated antimouse IgG secondary antibody (Merck Millipore, Bedford, MA) at room temperature for an additional hour, rinsed 3 times with PBS for 5 minutes each, and mounted with a mounting medium containing 4,6-diamidino-2-phenylindole (DAPI).

### Statistical analysis

For statistical analysis, SPSS software (Version 14.0, Chicago, IL) was used. Each sample was measured in triplicated, and data were expressed as mean ± standard error of the mean (SEM) (rat, n = 6 for each group). Tissue cytokine and Western blot measurements were tested using one-way analysis of variance and Tukey pairwise comparison with *P* < 0.05 representing statistical significance.

## Results

### Fat grafts ameliorates burn-induced neuropathic pain

Burn-induced hindpaw skin inflammations caused mechanical allodynia, and the PWT (g) of rats significantly decreased. The burns elicited mechanical allodynia. No significant change occurred in the PWL(s) and PWT (g) observations in the sham group or in the sham burn with fat graft treatment group (Fig 1B and 1C). A marked decrease in the PWT following burn injuries was noted 1 week after the burn injuries. After the fat graft injection to the hind paw skin, the PWT (g) of rat increased significantly in rats compared with the burn with saline injection group at 5, 6, 7, and 8 weeks. In other words, the anti-nociceptive effects of the fat grafts were strikingly different from 5 to 8 weeks after the burn injuries compared with Group C. Taken together; the results demonstrated that fat grafts ameliorated burn-induced pain such as mechanical allodynia.

### Fat grafts reduce COX-2, iNOS, and nNOS in immunohistochemical localization and attenuates protein expressions in the hind paw skin

**Fig 2:**
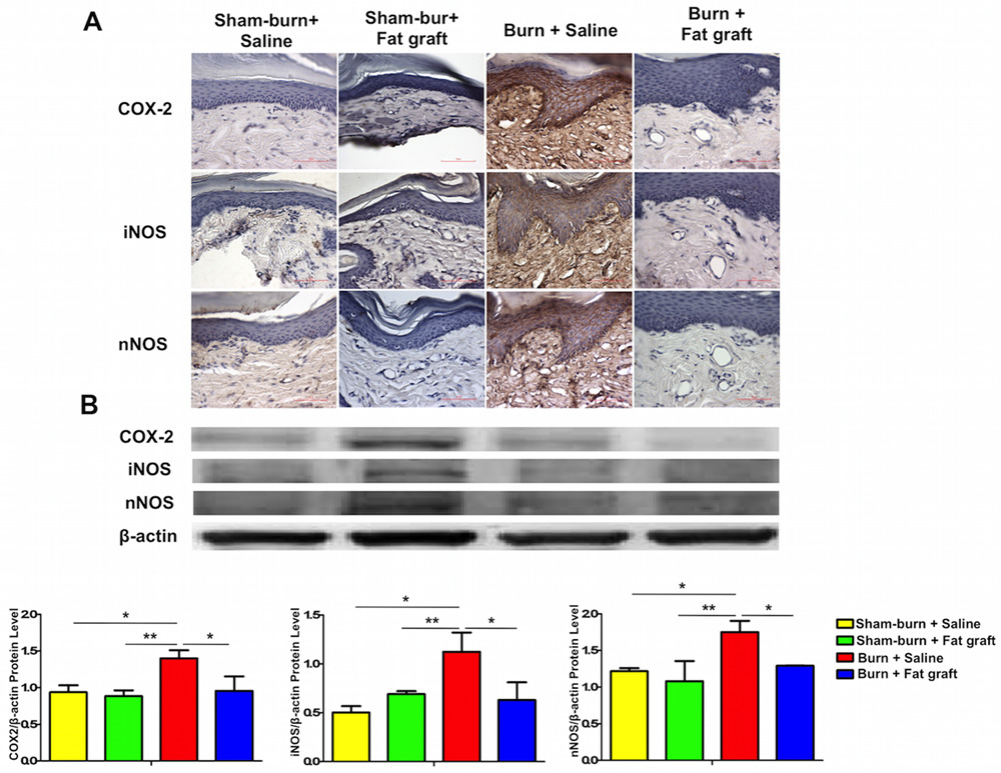
IHC staining images and protein expressions of COX-2, iNOS, and nNOS in the hind paw skin. (**A**) The effect of fat grafts on the expression of the inflammatory proteins,COX-2, iNOS and nNOS in the hind paw skin induced by a third-degree burn. (**B**) Western blot analyses of COX-2, iNOS and nNOS in the hind paw skin at week-8 after the burn injury. (Above) Western blot images were illustrated, and β**-**actin was used as the internal control. (Bottom) Quantitative result analyses were demonstrated, and the sample size was n > 3 for each test. The expressions of COX-2, iNOS and nNOS were decreased significantly in the burn with fat graft (Group D) compared with the burn with saline injection (Group C). *P < 0.05.

IHC staining images (Fig 2A) and protein expressions (Fig 2B) further confirmed that COX-2, iNOS, and nNOS activations were involved in the hind paw skin after the burn injuries. We found that the burns induced an increase of COX-2, iNOS, and nNOS protein levels in the hind paw skin 8 weeks after injury. Fat grafts significantly attenuated burn-induced COX-2, iNOS, and nNOS protein levels 4 weeks after treatment.

### The inflammatory COX-2, iNOS, and nNOS proteins and pro-inflammatory cytokines in the spinal cord dorsal horns are significantly attenuated after fat graft injection

**Fig 3:**
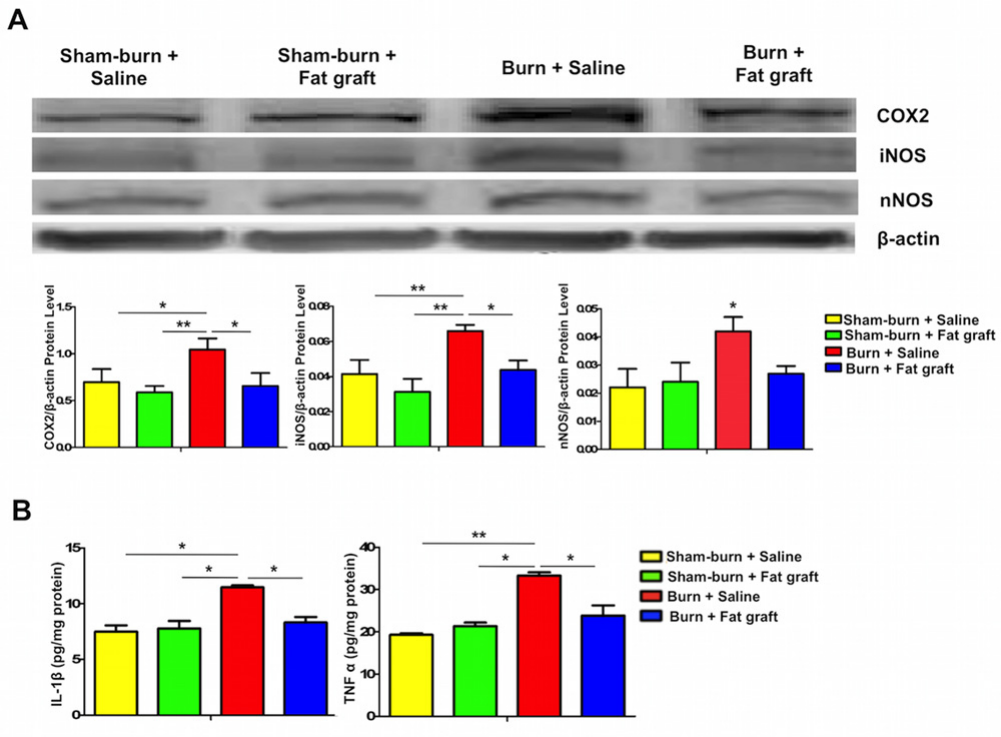
Fat grafting inhibited the expressions of inflammatory proteins, COX-2, iNOS and nNOS, in the spinal cord dorsal horn, and reduced the secreted levels of pro-inflammatory cytokines, IL-1β and TNF-α. (**A**) Western blot analyses of COX-2, iNOS and nNOS in the spinal cord dorsal horn at 8 weeks after the burn injury. β**-**actin was used as the internal control. The presentation format is similar to Fig 2. Protein expressions of COX-2, iNOS and nNOS were decreased significantly in the burn with fat graft group compared with the burn with saline injection group (Group D vs. Group C). (**B**) Fat grafts appreciably reduced the secretions of the pro-inflammatory cytokines, IL-1β and TNF-α, in the spinal cord dorsal horn. IL-1β and TNF-α assessment was conducted at least in triplicate, n > 3, *P < 0.05.

Burns induced the expression of several inflammatory proteins in the spinal cord dorsal horns. COX-2, iNOS, and nNOS were shown 8 weeks after the burn injuries compared with Group A, Group B and Group D. The fat grafting in the Group B showed no significant effects on these proteins compared with Group A. The fat grafts significantly attenuated inflammatory proteins in the spinal cord dorsal horns compared with Group D (Fig 3A). To explore the possible mechanism of the fat grafting effect on burn-induced neuropathic pain, we quantified the levels of IL-1β and TNF-α in the spinal cord dorsal horns (Fig 3B). We found significant neuropathic pain in paw withdrawal latency and inflammatory proteins in the spinal cords after burn injury. Therefore, pro-inflammatory cytokines were assessed; IL-1β and TNF-α levels were markedly decreased in Group D compared with Group C. Fat grafting thus alleviated neuroinflammation in the burn-induced neuropathic pain.

### Fat grafts increased p-IκB, reduced the p-NFκB expression and attenuated apoptosis in the spinal cord dorsal horns

**Fig 4:**
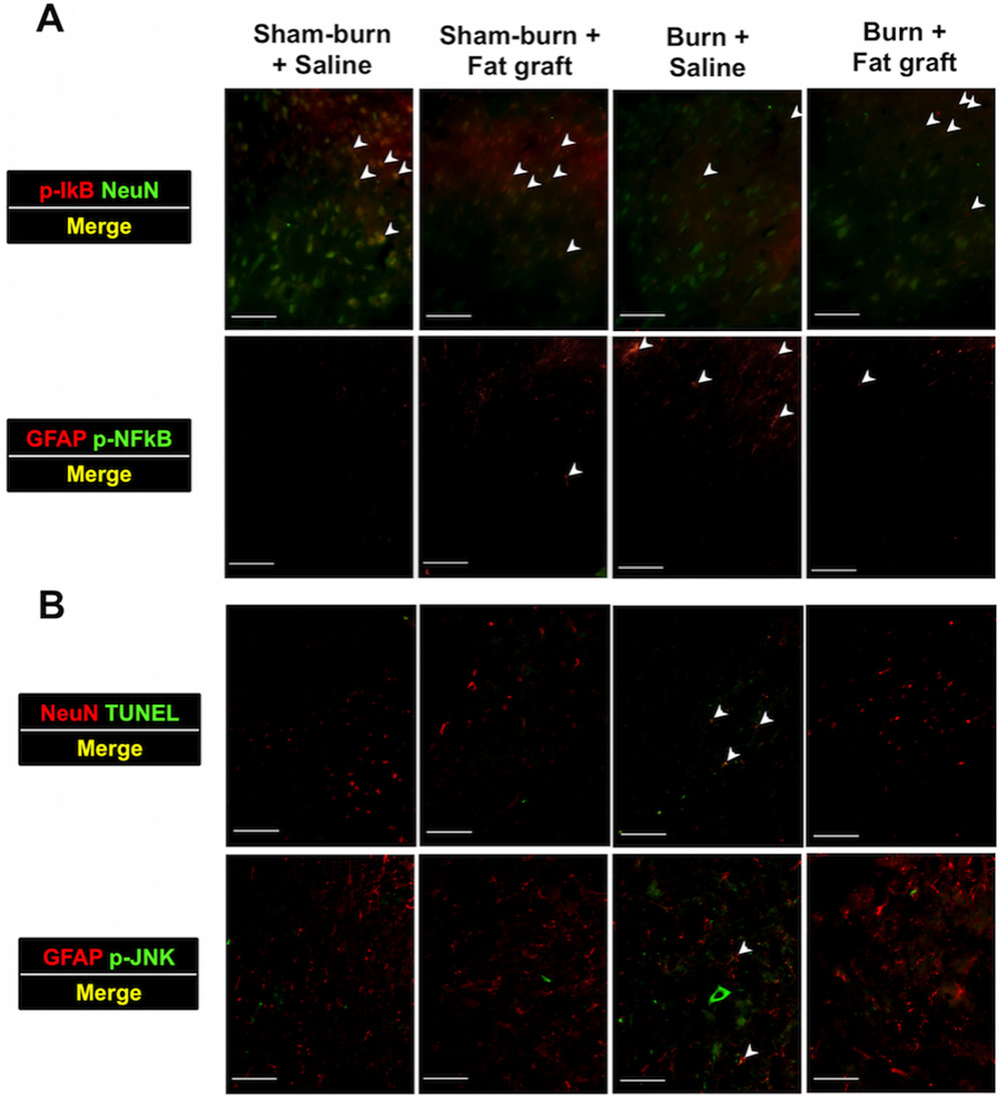
Fat grafts increased p-IκB, reduced the p-NFκB protein, and through the inhibition of p-JNK, attenuated apoptosis in the spinal cord dorsal horn. (**A**) Double immunofluorescent staining was performed to measure p-IκB and p-NFκB. Few p-IκB proteins were expressed in Group C, but p-IκB were increased in Group D. The expression of p-NFκB was abolished and reduced by fat grafts in Group D versus Group C. (**B**) Double immunofluorescence images in p-JNK and TUNEL expression were elevated following an increase in the TUNEL-positive cells. In Group D, we found that the inhibition from fat graft of p-JNK attenuated apoptosis in the spinal cord dorsal horn.

To reveal p-NFκB activation and neuron cell apoptosis in the spinal cords, we performed double immunofluorescent staining to measure p-IκB (blocking NFκB activation), p-NFκB (an inflammation marker) followed by p-JNK (an inflammation marker), and TUNEL (an apoptosis marker) (Fig 4). The total number of NeuN and p-IκB double positive cells in Group C decreased significantly compared with other groups. Additionally, the number of p-NFκB-expressing astrocytes was significantly higher in Group C than in Group D. Double immunofluorescent staining for p-JNK and TUNEL was performed in the spinal dorsal horns 4 weeks after fat grafts or saline injections. P-JNK and TUNEL double positive cells were significantly decreased in the spinal dorsal horn in Group D compared with Group C.

### Fat grafts decreased the p-AKT/AKT and Bax/Bcl-2 ratios in the spinal cord dorsal horns

**Fig 5:**
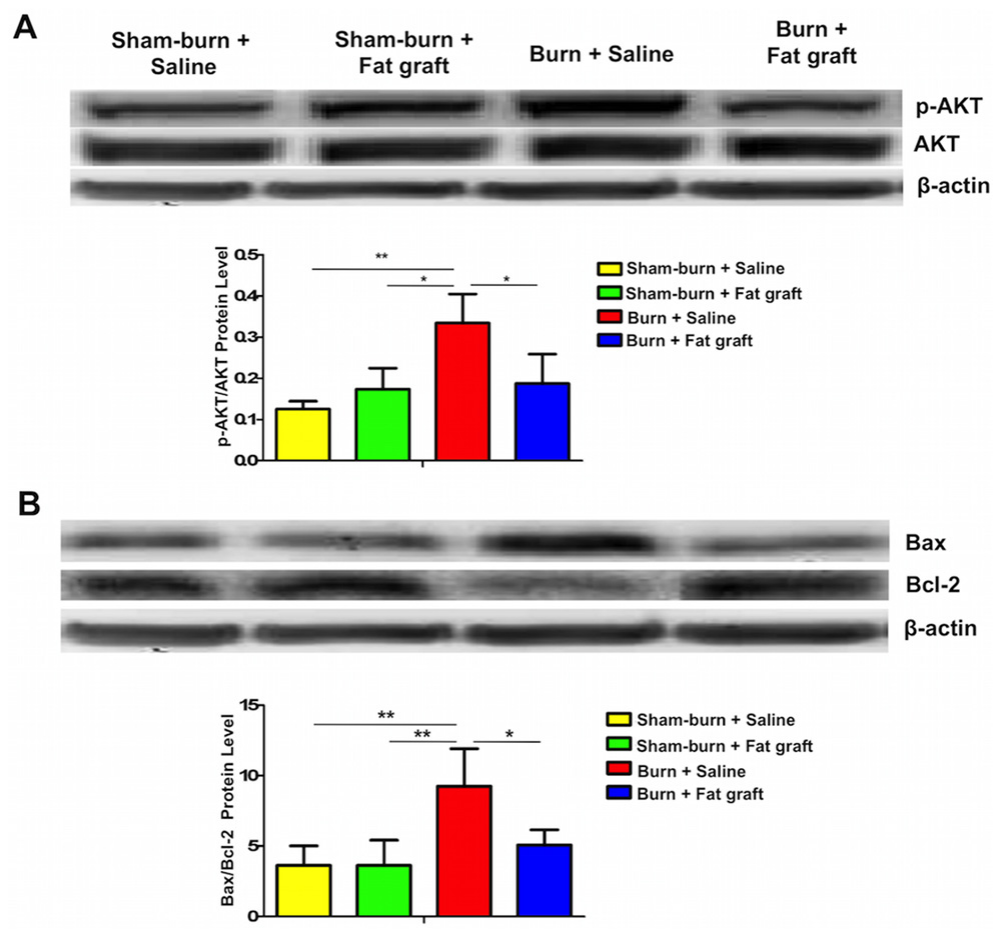
Fat graft regulated p-AKT/AKT and Bax/Bcl-2 ratios in the spinal cord dorsal horn. (**A** and **B**) Both expression ratios of p-AKT/AKT and Bax/Bcl-2 increased significantly in Group C versus Group D. These results indicated that fat grafting reduced cell death of neuron in the spinal cord (Data were presented with mean ± SEM, **P < 0.01; *P < 0.05).

The p-AKT/AKT and Bax/Bcl-2 expressions were assessed by Western blotting. The expression of 42 kb β-actin was used as a loading control. Group D significantly decreased both in p-AKT/AKT and Bax/Bcl-2 ratios compared with Group C. An increase in the Bax downstream processes of apoptosis in the spinal cord dorsal horn was also observed (Fig 5).

## Discussion

Fat grafts alleviate burn-induced neuropathic pain or traumatic painful scar had been reported, but the mechanism of fat graft in neuropathic pain is still not been elucidated [4, 5]. In this study, we have demonstrated that fat grafting is a promising method for alleviated burn-induced neuropathic pain, fat grafts reduce skin inflammation and neuroinflammation in the spinal cord further diminishes neuron cell apoptosis. Furthermore, fat graft also reduced inflammation protein expression such as effect iNOS, nNOS, COX-2 in burn-injured skin as well as in spinal cord dorsal horn.

Ipaktchi et al. reported that burn would induce excessive local inflammation and producing inflammatory cytokines [27]. Furthermore, burn wounds and their healing processes can cause inflammatory mediators and cells, such as T-helper cells and macrophages. In the wound healing process, pro-inflammatory mediators, such as TNF-α, TGF-β, and the interleukin family are highly up-regulated [28]. Therefore, inflammation is a physiologic phenomenon of wound healing that may occur for over 12 months until scars mature [29]. Inflammation and nerve injuries induce transcriptional change and the induction of COX-2 in dorsal horn neurons [30]. This causes burn scar neuropathic pain, and if inflammation persists, it also causes second necrosis and apoptosis in the spinal cord [31].

In addition to the inflammatory induction of COX-2, which leads to the release of prostanoids and localized pain hypersensitivity, this local inflammation also induced pain in uninjured area by increasing neuronal excitability in the central nerve system thus as spinal cord (also known as central sensitization). Samad et al demonstrated that in the hind paw inflammation model evoked by complete Freund’s adjuvant, the hind paw skin produced high increases in COX-2 mRNA and a similar increase in the lumbar spinal cord. The inhibition of IL-1β signaling prevents transcriptional upregulation of the COX-2 gene, reduced mechanical hyperalgesia, and normalized pain sensitivity [32]. The pro-inflammatory cytokines, IL-1β and TNF-α, were key cytokines during inflammation and reduced mechanical pain thresholds. Moreover, pro-inflammatory cytokine antagonists were able to reduce hyperalgesia in the inflammation model, meaning that the activation of IL-1β and TNF-α was a crucial step in the generation of inflammatory pain [25]. Our data showed that fat grafting decreased the expression of COX-2 in IHC staining and in western blot in skin and spinal cord dorsal horn. Furthermore, fat grafting decreased the level of IL-1β and TNF-α in the spinal cord dorsal horn. Therefore, we suggested that fat grafting alleviated burn-induced neuropathic pain by decreasing the level of IL-1β and further reduced the expression of COX-2 to alleviate the inflammation process.

The overexpression or inappropriate NO produced by iNOS and/or nNOS is associated with inflammatory and neuropathic pain. Upregulated nNOS and iNOS expressions are noted in the spinal cord and skin after nerve or burn injuries in neuropathic pain animal models [33, 34]. Payne et al demonstrated that iNOS/nNOS inhibitors were effective in the model of neuropathic pain [35]. The inhibition of NOS in the neuropathic pain model diminishes the upregulation of iNOS and nNOS in the spinal cord and skin. Therefore, iNOS and nNOS can indicate neuropathic pain. Keihoff et al demonstrated the time-course of neuropathic pain in chronic constriction injury (CCI) model, in wild-type mice, the neuropathic pain was noted in day 10 after chronic constriction injury (CCI) and was normalized on day 52. However, the pain threshold normalized on day 6 in the nNOS- knockout mice and day 17 in the iNOS mice. This resulted from the required regulatory role of iNOS in the upregulation of nNOS [22, 36]. In our results, fat grafts alleviated allodynia in the behavior test, decreased iNOS and nNOS expression in the IHC staining images and decreased protein expression. Additionally, an increased protein expression was shown in the spinal cord dorsal horn. These results indicated that fat grafting alleviated the skin and spinal cord neuroinflammatory reaction and improved the neuropathic pain.

NFκB is a nuclear transcription factor that regulates inflammation and apoptosis. Activation of the NFκB signal pathway depends on phosphorylation and degradation of IκB proteins [37]. The MAPKs play essential regulatory roles in neuronal inflammatory response [38,39]. A recent study indicated that the JNK pathway mediated apoptosis involving an inflammatory response in the neuroblastoma cell line [40]. This apoptosis reaction was also revealed in the expression of AKT and the increasing of the Bax/Bcl-2 ratio [41, 42]. Activation of phosphatidylinositol 3 kinase/AKT has been linked to cytotoxic cell death, and AKT inhibitors also protect cultured neurons against photodynamic-induced necrosis [43]. Bax and Bcl-2 play central roles in regulating apoptosis. The decreasing expression of Bax and the increasing expression of Bcl-2 promote cell survival by inhibiting apoptosis [42]. Our data revealed that fat grafting may increase p-IκB and decrease the NFκB transcription in anti-inflammation. Through the inhibition of AKT and JNK phosphorylation in astrocytes, decreasing the expression of Bax and increasing the expression of Bcl-2 to alleviate apoptosis in the spinal cord dorsal horn thus alleviates apoptosis [43,44]. We speculated that fat grafting alleviated neuropathic pain through the anti-inflammatory effect and ameliorated the apoptosis in the spinal cord dorsal horn neuron cells.

## Conclusions

The subcutaneous injection of fat grafts in burn-injured hind paw skin significantly attenuated burn-induced neuropathic pain. In summary, our results revealed that fat grafting reduced p-NFκB activation in spinal astrocytes and attenuated inflammatory proteins including COX-2, iNOS, and nNOS in spinal cord and burn scar. Our findings thus showed that fat grafting, because of its anti-inflammatory effect, can be used as an innovative therapeutic method for burn-induced neuropathic pain.
